# Microwave-assisted one pot three-component synthesis of some novel pyrazole scaffolds as potent anticancer agents

**DOI:** 10.1186/s13065-017-0266-4

**Published:** 2017-05-08

**Authors:** Sobhi M. Gomha, Mastoura M. Edrees, Rasha A. M. Faty, Zeinab A. Muhammad, Yahia N. Mabkhot

**Affiliations:** 1 Department of Chemistry, Faculty of Science, Cairo University, Giza, 12613 Egypt; 2 Department of Organic Chemistry, National Organization for Drug Control and Research (NODCAR), Giza, 12311 Egypt; 3 Faculty of Science, King Khalid University, Abha, Kingdom of Saudi Arabia; 4 Department of Chemistry, College of Science, King Saud University, P. O. Box 2455, Riyadh, 11451 Kingdom of Saudi Arabia

**Keywords:** Acetylpyrazoles, Enaminones, Hydrazonoyl chlorides, Thiazoles, Thiadiazoles, Anticancer activity

## Abstract

**Background:**

Pyrazoles, thiazoles and 1,3,4-thiadiazoles have been reported to possess various pharmacological activities.

**Results:**

An efficient and a novel approach for the synthesis of some novel pyrazole based-azoles are described via multi-component reaction under controlled microwave heating conditions. The structures of the synthesized compounds were assigned on the basis of elemental analysis, IR, ^1^H NMR and mass spectral data. All the synthesized compounds were tested for in vitro activities against two antitumor cell lines, human lung cancer and human hepatocellular carcinoma compared with the employed standard antitumor drug (cisplatin).

**Conclusions:**

All the newly synthesized compounds were evaluated for their anticancer activity against human lung cancer and human hepatocellular carcinoma cell lines using MTT assay. The results obtained exploring the high potency of six of the tested compounds compared with cisplatin.

**Graphical abstract:**
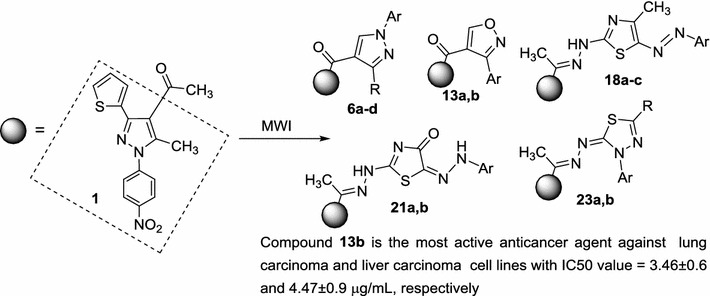
Microwave-assisted one pot three-component synthesis of some novel pyrazole scaffolds as potent anticancer agents

## Background

Multi-component reactions (MCR) are one-pot processes with at least three components to form a single product, which incorporates most or even all of the starting materials [1–6]. The huge interest for such multi-component reactions during the last years has been oriented towards developing combinatorial chemistry procedures, because of their high efficiency and convenience of these reactions in comparison with multistage procedures. Also, the utility of MCR under microwave irradiation in synthesis of heterocyclic compounds enhanced the reaction rates and improve the regioselectivity [7–12].

On the other hand, pyrazole and its derivatives have drawn considerable attention of the researchers in the past few decades owing to their high therapeutic values. Some of the drugs, possessing pyrazole as basic moiety, like celecoxib [13], deracoxib [14], etoricoxib and atorivodine [15] are already booming in the market. Pyrazole derivatives possess an extensive range of pharmacological activities such as antiinflammatory, antipyretic, analgesic, antimicrobial, sodium channel blocker, antitubercular, antiviral, antihypertensive, antiglaucoma, antioxidant, antidepressant, anxiolytic, neuroprotective and antidiabetic activity [16–23]. Furthermore, pyrazole prodrugs have also been reported to possess significant anticancer activities [24–30]. Keeping this in mind, and in continuation of our previous work on the synthesis of new anticancer agents [31–40], we herein present an efficient regioselective synthesis of novel 4-heteroaryl-pyrazoles, which have not been reported *hitherto* in a multicomponent synthesis under microwave irradiation and to assess their anticarcinogenic effects against hepatocellular carcinoma (HepG-2) and human lung cancer (A-549) cell lines.

## Results and discussion

### Chemistry

**Scheme 1:**
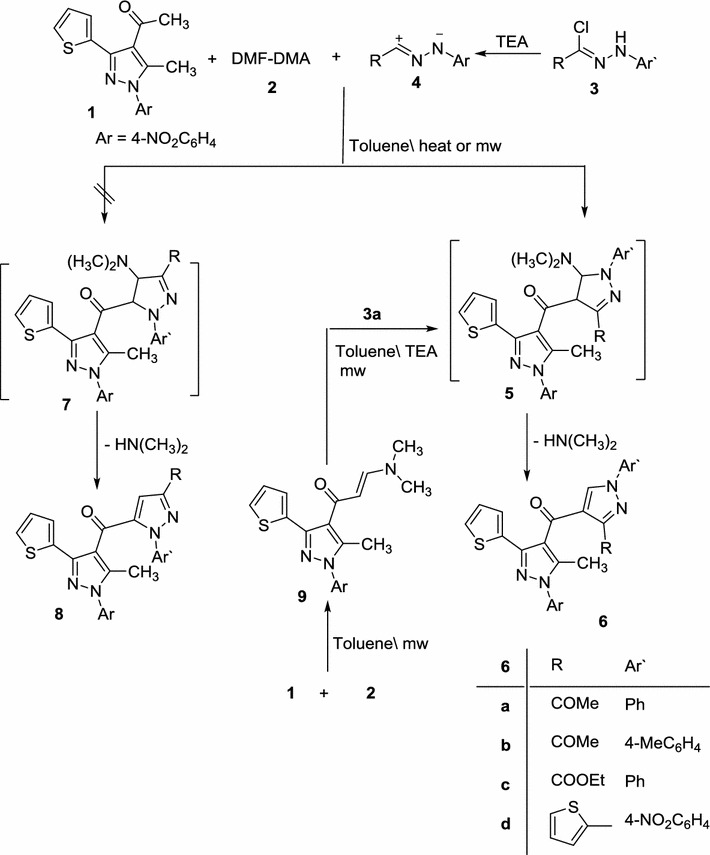
Synthesis of pyrazoles **6a**–**d**

Multi-component reaction of acetyl pyrazole **1** [41], dimethylformamide dimethylacetal (DMF–DMA) **2** and nitrileimine **4a**–**d** (generated in situ from **3a**–**d** with triethylamine) in toluene under conventional heating for 10–15 h or under microwave irradiation at 150 °C for 4–10 min. afforded compound **6a**–**d** rather than its isomeric structure **8a**–**d** in 66–70 and 84–90%, respectively (Scheme 1; Table 1). The structure of **6a**–**d** was confirmed by their spectral data (IR, MS and ^1^H-NMR) and elemental analyses. For example, the IR spectra of products **6** revealed in each case two absorption bands in the regions υ 1638–1676 and 1682–1724 cm^−1^ due to the two carbonyl groups. The ^1^HNMR spectra showed, in addition to the expected signals for the aromatic protons, three singlet signals at *δ* ~2.34, 2.55 and 8.92 reveled to the two methyl groups and the pyrazole-H5, respectively. The mass spectra of products **6a**–**d** revealed a molecular ion peak for each one which is consistent with the respective molecular weight. These data are much closer to those reported in literature on similar work [42–44].

**Table 1:** Comparative data of conventional (A) and MW (B) methods for the synthesis of compounds **6a**–**d**, **13a**, **b**, **18a**–**c**, **21a**, **b** and **23a**, **b**

Compound no.	Conventional method (A)		Microwave method (B)	
	Time (h)	Yield (%)	Time (min)	Yield (%)
**6a**	12	66	4	84
**6b**	15	68	10	85
**6c**	10	70	8	88
**6d**	8	69	5	90
**13a**	12	67	6	82
**13b**	10	70	6	89
**18a**	8	66	7	90
**18b**	6	68	10	88
**18c**	4	67	7	90
**21a**	6	69	8	86
**21b**	5	64	6	92
**23a**	8	72	10	81
**23b**	8	67	9	83

Compound **6a** was alternatively synthesized by reacting enaminone **9** (prepared separately via condensation of acetyl pyrazole **1** with DMF–DMF) with 2-oxo-*N*-phenylpropanehydrazonoyl chloride (**3a**) in toluene containing catalytic amount of TEA under MWI. The obtained product was found to be identical with **6a** in all respects (TLC, mp and IR spectrum) which affords further evidence to all structures **6a**–**d**. The latter products were assumed to be formed via initial 1,3-dipolar cycloaddition of the nitrileimines **4a**–**d** to the activated double bond in enaminone **9** to afford the non-isolable cycloadducts **5** which underwent loss of dimethylamine yielding the final pyrazole derivatives **6a**–**d**.

The results obtained Table 1 indicate that, unlike classical heating, microwave irradiation results in higher yields and shorter reaction times for all the carried reactions. Microwave irradiation facilitates the polarization of the molecules under irradiation causing rapid reaction to occur. This is consistent with the reaction mechanism, which involves a polar transition state [45].

**Scheme 2:**
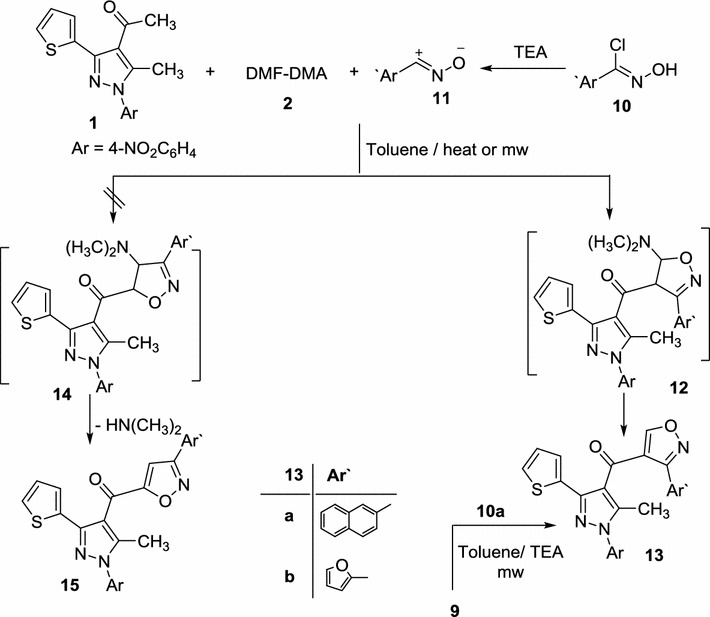
Synthesis of isoxazoles **13a**, **b**

By the same way reaction of acetyl pyrazole **1** with nitrile-oxide **11a**, **b** (derived from reaction of hydroximoyl chloride **10a**, **b** with TEA) and DMF–DMA in toluene under microwave irradiation at 150 °C gave isoxazoles **13a**, **b** (Scheme 2; Table 1). The ^1^H NMR spectrum of the product revealed a singlet signal at 9.67 ppm assigned for isoxazole-5H proton not isoxazole-4H proton [42–44, 46] which consistent with the isomeric structure **13** rather than the isomeric structure **15**. Moreover, the mass spectrum of **13a** and **13b** revealed a molecular ion peaks at m/z = 506 and 446, respectively, which is consistent with their molecular weights.

Furthermore, alternative synthesis of compound **13a** was achieved via reaction enaminone **9** with *N*-hydroxy-2-naphthimidoyl chloride (**10a**) under the same reaction condition to yield authentic product **13a** (Scheme 2).

**Scheme 3:**
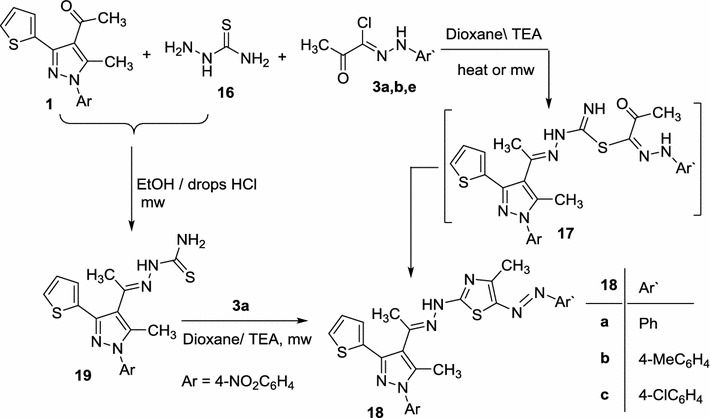
Synthesis of thiazoles **18a**–**c**

Next, our study was extended to investigate the reactivity of compound **1** towards thiosemicarbazide and various hydrazonoyl halides aiming to synthesize new pyrazole based—1,3-thiazoles and 1,3,4-thiadiazoles. Thus, acetyl pyrrole **1**, thiosemicarbazide **2** and α-keto hydrazonoyl halides **3a**, **b**, **e** were allowed to react in a one-pot three-component reaction in dioxane containing catalytic amount of TEA under MWI to afford the arylazothiazole derivatives **18a**–**c**, respectively (Scheme 3; Table 1). The reaction goes in parallel to literature [32, 35–37].

The structure of the products **18a**–**c** was assigned based on the spectral data and elemental analyses. For example mass spectrum of compound **18a** revealed molecular ion peak at m/z 542 and its ^1^H NMR spectrum exhibited four characteristic singlet signals at 2.32, 2.36, 2.48 and 10.47 assignable to three CH_3_ groups and NH protons, respectively, in addition to an aromatic multiplet in the region 6.99–7.93 ppm equivalent to 12 protons. Its IR spectra showed one NH group band at 3396 cm^−1^.

The structure of products **18** was further confirmed by an alternative method. Thus, reaction of acetylpyrazole **1** with thiosemicarbazide **16** under MWI in ethanol containing drops of concentrated HCl led to the formation of product **19**. Compound **19** was then react with 2-oxo-*N*-phenylpropanehydrazonoyl chloride (**3a**) in dioxane containing catalytic amount of TEA under MWI to give a product identical in all respects (IR, mp and mixed mp.) with **18a** (Scheme 3).

**Scheme 4:**
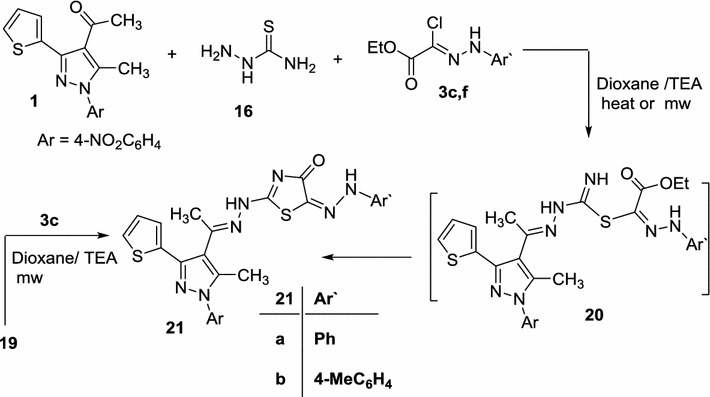
Synthesis of thiazolones **21a**, **b**

In a similar manner, when acetyl pyrazole **1** was allowed to react with thiosemicarbazide **2** and ethyl (*N*-arylhydrazono)-chloroacetates **3c**, **f** in dioxane in the presence of triethylamine under MWI, it afforded in each case a single isolable product, namely, 2-(2-(1-(5-methyl-1-(4-nitrophenyl)-3-(thiophen-2-yl)-1*H*-pyrazol-4-yl)ethylidene) hydrazinyl)-5-(2-arylhydrazono) thiazol-4(5*H*)-one **21a**, **b** (Scheme 4; Table 1). Structure **21** was confirmed by elemental analysis, spectral data (IR, ^1^H NMR, and mass), and alternative synthesis route. Thus, thiosemicarbazone **19** was reacted with ethyl)-2-chloro-2-(2-phenylhydrazono)acetate (**3c**) in dioxane in the presence of TEA under MWI afforded a product identical in all aspects (mp, mixed mp, and spectra) with **21a** (Scheme 4).

**Scheme 5:**
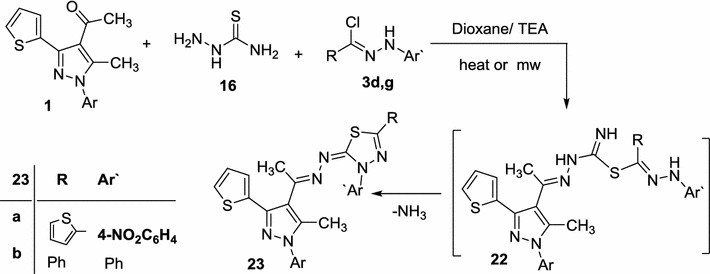
Synthesis of thiadiazoles **23a**, **b**

Finally, the reactivity of acetylpyrazole **1** towards hydrazonoyl halides, be bereft of a-keto group, was examined. In the present study, we have established that reaction of acetylpyrazole **1** with *N*-thiosemicarbazide **16** and aryl carbohydrazonoyl chlorides **3d**, **g** gave the respective 1,3,4-thiadiazoles **23a**, **b** as the end products (Scheme 5; Table 1). The structures of compounds **23a**, **b** were confirmed on the bases of spectral data and elemental analyses (see Experimental part). The reaction proceeded via S-alkylation, with removal of hydrogen chloride, to give S-alkylated intermediates **22** followed by intramolecular Michael type addition under the employed reaction conditions, followed by elimination of ammonia, afforded the final product **23** [36, 47] (Scheme 5).

### Cytotoxic activity

**Fig. 1:**
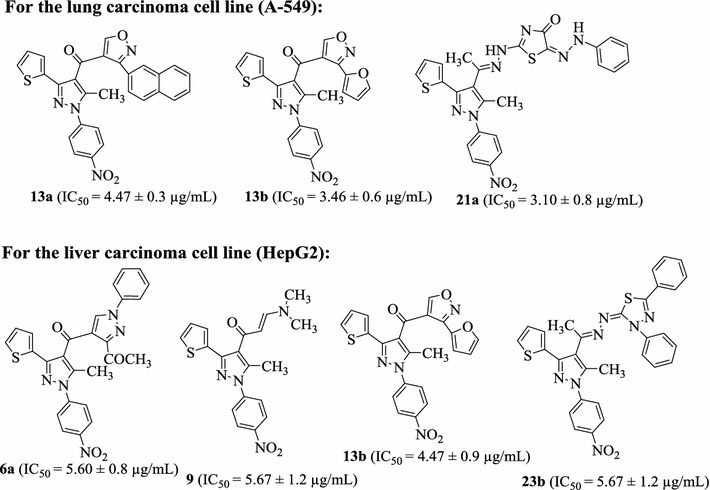
Cytotoxic activities of the most active compounds against HEPG2 and A-549 cell lines

The in vitro growth inhibitory activity of the synthesized compounds **6a**–**d**, **9**, **13a**, **b**, **18a**–**c**, **19**, **21a**, **b** and **23a**, **b** was investigated against two carcinoma cell lines: human lung cancer (A-549) and human hepatocellular carcinoma(HepG-2) in comparison with the well-known anticancer standard drug (cisplatin) under the same conditions using colorimetric MTT assay. Data generated were used to plot a dose response curve of which the concentration of test compounds required to kill 50% of cell population (IC_50_) was determined. The results revealed that all the tested compounds showed inhibitory activity to the tumor cell lines in a concentration dependent manner. Interestingly, the results represented in Table 2 and Fig. 1 showed that compounds **13a**, **13b** and **21a** were the most active compounds (IC_50_ value of 4.47 ± 0.3, 3.46 ± 0.6, 3.10 ± 0.8 μg/mL, respectively) against the lung carcinoma cell line (A549), compared with cisplatin reference drug with IC_50_ value of 0.95 ± 0.23 μg/mL. Moreover, the order of activity against A549 cell line was **18c** > **18b** > **19** > **9** > **6a** > **6c** > **23b** > **6d** > **18a** > **21b** > **6b**.

**Table 2:** The in vitro inhibitory activity of tested compounds against tumor cell lines expressed as IC50 values (μg/mL) ±standard deviation from three replicates

Tested compounds	R	Ar′	Tumor cell lines	
			A-549	HepG2
**6a**	COCH_3_	Ph	22.9 ± 0.9	5.60 ± 0.8
**6b**	COCH_3_	4-MeC_6_H_4_	38.5 ± 1.2	44.4 ± 1.3
**6c**	COOEt	Ph	23.3 ± 0.9	22.4 ± 0.9
**6d**	2-Thienyl	4-NO_2_C_6_H_4_	30.6 ± 1.1	35.9 ± 1.4
**9**	–	–	22.6 ± 0.8	5.67 ± 1.2
**13a**	–	2-Naphthyl	4.47 ± 0.3	8.03 ± 1.1
**13b**	–	2-Furyl	3.46 ± 0.6	4.67 ± 0.9
**18a**	–	Ph	32.7 ± 1.2	22.4 ± 1.1
**18b**	–	4-MeC_6_H_4_	19.1 ± 1.1	6.67 ± 1.3
**18c**	–	4-ClC_6_H_4_	18.2 ± 0.9	21.8 ± 0.9
**19**	–	–	21.3 ± 0.8	23.1 ± 1.1
**21a**	–	Ph	3.10 ± 0.8	23.9 ± 1.1
**21b**	–	4-MeC_6_H_4_	33.6 ± 0.9	43.4 ± 0.8
**23a**	2-Thienyl	4-NO_2_C_6_H_4_	27.9 ± 1.1	34.4 ± 0.9
**23b**	Ph	Ph	23.4 ± 1.2	5.67 ± 1.7
**Cisplatin**	–	–	0.95 ± 0.23	1.4 ± 0.37

On the other hand, compounds **6a**, **9**, **13b**, **23b** were the most active compounds (IC_50_ value of 5.60 ± 0.8, 5.67 ± 1.2, 4.47 ± 0.9 and 5.67 ± 1.2 μg/mL, respectively) against liver carcinoma cell line (HepG2) cell line while the rest compounds have moderate activities.

## Experimental

### Chemistry

#### General

Melting points were measured on an Electrothermal IA 9000 series digital melting point apparatus (Bibby Sci. Lim. Stone, Staffordshire, UK). IR spectra were measured on PyeUnicam SP 3300 and Shimadzu FTIR 8101 PC infrared spectrophotometers (Shimadzu, Tokyo, Japan) in potassium bromide discs. NMR spectra were measured on a Varian Mercury VX-300 NMR spectrometer (Varian, Inc., Karlsruhe, Germany) operating at 300 MHz (^1^H-NMR) and run in deuterated dimethylsulfoxide (DMSO-*d*_*6*_). Chemical shifts were related to that of the solvent. Mass spectra were recorded on a Shimadzu GCMS-QP1000 EX mass spectrometer (Tokyo, Japan) at 70 eV. Elemental analyses were measured by using a German made Elementarvario LIII CHNS analyzer. Antitumor activity of the products was measured at the Regional Center for Mycology and Biotechnology at Al-Azhar University, Cairo, Egypt. Hydrazonoyl halides **3a**–**g** were prepared following literature method [41, 48].

#### Synthetic procedures

##### Synthesis of trisubstituted pyrazoles 6a-d and isoxazoles 13a,b

*Method A* To a stirred solution of acetyl pyrazole **1** (0.327 g, 1 mmol), dimethylformamide dimethylacetal **2** (1 mmol) and the appropriate hydrazonoyl halides **3a**–**d** or hyroximoyl chlorides **10a**, **b** (1 mmol) in dry toluene (15 mL), an equivalent amount of triethylamine (0.5 mL) was added. The reaction mixture was heated under reflux for 10–15 h (monitored through TLC). The precipitated triethylamine hydrochloride was filtered off, and the filtrate was evaporated under reduced pressure. The residue was triturated with MeOH. The solid product, so formed in each case, was collected by filtration, washed with water, dried, and crystallized from the proper solvent to afford the corresponding pyrazole **6a**–**d** and isoxazole derivatives **13a**, **b**, respectively.

##### Method B

Repetition of the same reactions of method A with heating in microwave oven at 500 W and 150 °C for 4–10 min., gave products identical in all respects with those separated from method A. The products **6a**–**d** and **13a**, **b** together with their physical constants are listed below.

##### 1-(4-(5-Methyl-1-(4-nitrophenyl)-3-(thiophen-2-yl)-1*H*-pyrazole-4-carbonyl)-1-phenyl-1*H*-pyrazol-3-yl)ethanone (**6a**)

Brown solid, mp 208–210 °C; IR (KBr) ν_max_ 1599 (C=N),1670, 1682 (2C=O), 2924, 3105 (C–H) cm^−1^; ^1^H NMR (DMSO-*d*_*6*_) *δ* 2.34 (s, 3H, CH_3_), 2.55 (s, 3H, CH_3_), 6.98–8.39 (m, 12H, Ar–H), 8.92 (s, 1H, pyrazole-H5); MS m/z (%) 497 (M^+^, 9), 342 (25), 252 (22), 174 (11), 145 (22), 115 (26), 103 (40), 76 (100), 63 (13), 50 (19). Anal. Calcd. for C_26_H_19_N_5_O_4_S (497.53): C, 62.77; H, 3.85; N, 14.08. Found: C, 63.08; H, 3.55; N, 13.70%.

##### 1-(4-(5-Methyl-1-(4-nitrophenyl)-3-(thiophen-2-yl)-1*H*-pyrazole-4-carbonyl)-1-(*p*-tolyl)-1*H*-pyrazol-3-yl)ethanone (**6b**)

Yellow solid, mp 222–224 °C; IR (KBr) ν_max_ 1597 (C=N),1676, 1688 (2C=O), 2919, 3118 (C–H) cm^−1^; ^1^H NMR (DMSO-*d*_*6*_) *δ* 2.24 (s, 3H, CH_3_), 2.34 (s, 3H, CH_3_), 2.56 (s, 3H, CH_3_), 7.12 (t, *J* = 1.2 Hz, 1H, thiophene-H), 7.31 (d, *J* = 1.2 Hz, 1H, thiophene-H), 7.33 (d, *J* = 1.2 Hz, 1H, thiophene-H), 7.55 (d, *J* = 4.4 Hz, 2H, Ar–H), 7.63 (d, *J* = 4.4 Hz, 2H, Ar–H),7.88 (d, *J* = 8.8 Hz, 2H, Ar–H), 8.39 (d, *J* = 8.8 Hz, 2H, Ar–H), 10.58 (s, 1H, pyrazole-H5); ^13^C-NMR (DMSO-*d*_6_): *δ* 13.3, 20.8, 25.7 (CH_3_), 115.3, 117.6, 118.9, 121.37, 122.7, 125.2, 126.7, 128.1, 129.4, 130.1, 132.2, 133.8, 138.1, 140.6, 143.43, 144.4, 146.8, 147.2 (Ar–C and C=N),188.2, 194.9 (C=O); MS m/z (%) 511 (M^+^, 2), 406 (10), 266 (6), 219 (11), 168 (7), 147 (7), 125 (11), 104 (25), 98 (17), 83 (93), 79 (44), 69 (35), 54 (53), 44 (100). Anal. Calcd. for C_27_H_21_N_5_O_4_S (511.55): C, 63.58; H, 4.14; N, 13.69. Found: C, 63.78; H, 4.05; N, 13.29%.

##### Ethyl 4-(5-methyl-1-(4-nitrophenyl)-3-(thiophen-2-yl)-1*H*-pyrazole-4-carbonyl)-1-phenyl-1*H*-pyrazole-3-carboxylate (**6c**)

Yellow solid, mp 207–209 °C; IR (KBr) ν_max_ 15,984 (C=N), 1660, 1724 (2C=O), 2931, 2974 (C–H) cm^−1^; ^1^H NMR (DMSO-*d*_*6*_) *δ* 1.18 (t, *J* = 7.6 Hz, 3H, CH_3_CH_2_), 2.34 (s, 3H, CH_3_), 4.27 (q, *J* = 7.1 Hz, 2H, CH_2_CH_3_), 6.96–8.43 (m, 12H, Ar–H), 8.99 (s, 1H, pyrazole-H5); MS m/z (%) 527 (M^+^, 6), 484 (22), 366 (26), 328 (33), 268 (50), 226 (35), 210 (37), 151 (49), 124 (78), 115 (61), 75 (100), 42 (45). Anal. Calcd. for C_27_H_21_N_5_O_5_S (527.55): C, 61.47; H, 4.01; N, 13.28. Found: C, 61.77; H, 3.75; N, 12.94%.

##### (5-Methyl-1-(4-nitrophenyl)-3-(thiophen-2-yl)-1*H*-pyrazol-4-yl)(1-(4-nitrophenyl)-3-(thiophen-2-yl)-1*H*-pyrazol-4-yl)methanone (**6d**)

Orange solid, mp 219–220 °C; IR (KBr) ν_max_ 1595 (C=N),1638 (C=O), 2924, 3105 (C–H) cm^−1^; ^1^H NMR (DMSO-*d*_*6*_) *δ* 2.34 (s, 3H, CH_3_), 6.98–8.52 (m, 14H, Ar–H), 9.28 (s, 1H, pyrazole-H5); ^13^C-NMR (DMSO-*d*_6_): *δ* 26.9 (CH_3_), 113.1, 113.3, 115.0, 115.6, 122.5, 122.6, 123.1, 123.6, 126.5, 126.7, 128.4, 131.1, 131.7, 132.1, 132.3, 136.5, 137.1, 141.5, 141.6, 142.4, 142.6, 142.8 (Ar–C and C=N), 197.2 (C=O); MS m/z (%) 582 (M^+^, 6), 532 (12), 383 (16), 286 (11), 219 (21), 135 (49), 79 (16), 83 (27), 76 (67), 60 (28), 45 (100). Anal. Calcd. for C_28_H_18_N_6_O_5_S_2_ (582.61): C, 57.72; H, 3.11; N, 14.42. Found: C, 57.99; H, 2.80; N, 14.12%.

##### Synthesis of 3-(dimethylamino)-1-(5-methyl-1-(4-nitrophenyl)-3-(thiophen-2-yl)-1*H*-pyrazol-4-yl)prop-2-en-1-one (**9**).

Amixture of acetyl pyrazole **1** (3.27 g, 10 mmol) and dimethylformamide–dimethylacetal (DMF–DMA) (10 mmol) in dry toluene (20 mL) was refluxed in microwave oven at 500 W and 150 °C for 5 min., then left to cool to room temperature. The precipitated product was filtered off, washed with light petroleum (40–60 °C), and dried. Recrystallization from benzene afforded enaminone **1** as orange solid, mp 250–252 °C; IR (KBr) ν_max_ 1642 (C=O), 2920, 3080 (C–H) cm^−1^; ^1^H NMR (DMSO-*d*_*6*_) *δ* 2.34 (s, 3H, CH_3_), 2.87 (s, 3H, CH_3_), 3.06 (s, 3H, CH_3_), 5.24 (d, *J* = 12.8 Hz, 1H, N–CH=), 7.05 (t, *J* = 1.2 Hz, 1H, thiophene-H), 7.14 (d, *J* = 1.2 Hz, 1H, thiophene-H), 7.50 (d, *J* = 1.2 Hz, 1H, thiophene-H), 7.65 (d, *J* = 12.8 Hz, 1H, =CH–CO), 7.90 (d, *J* = 8.8 Hz, 2H, Ar–H), 8.37 (d, *J* = 8.8 Hz, 2H, Ar–H); ^13^C-NMR (DMSO-*d*_6_): *δ* 12.4, 36.1, 44.0 (CH_3_), 120.4, 124.3, 124.4, 125.8, 127.0, 127.1, 128.4, 134.0, 142.5, 143.5, 145.4, 145.8, 146.3 (Ar–C and C=N), 194.0 (C=O); MS m/z (%) 382 (M^+^, 3), 300 (11), 286 (11), 189 (9), 132 (7), 104 (100), 77 (58), 64 (16), 51 (13), 43 (12). Anal. Calcd. for C_19_H_18_N_4_O_3_S (382.44): C, 59.67; H, 4.74; N, 14.65. Found: C, 59.58; H, 4.44; N, 14.39%.

##### (5-Methyl-1-(4-nitrophenyl)-3-(thiophen-2-yl)-1*H*-pyrazol-4-yl)(3-(naphthalen-2-yl)isoxazol-4-yl)methanone (**13a**)

Yellow solid, mp 203–205 °C; IR (KBr) ν_max_ 1597 (C=N), 1660 (C=O), 2976, 3117 (C–H) cm^−1^; ^1^H NMR (DMSO-*d*_*6*_) *δ* 2.31 (s, 3H, CH_3_), 7.13–8.45 (m, 14H, Ar–H), 9.67 (s, 1H, isoxazole-H5); ^13^C-NMR (DMSO-*d*_6_): *δ* 26.9 (CH_3_), 110.0, 113.3, 115.0, 115.1, 115.5, 122.5, 123.3, 124.5, 125.0, 126.5, 126.7, 128.4, 130.8, 133.6, 135.4, 136.9, 137.0, 141.5, 141.6, 142.6, 148.8, 152.4, 160.0 (Ar–C and C=N), 188.3 (C=O); MS m/z (%) 506 (M^+^, 2), 435 (9), 412 (14), 379 (45), 214 (12), 142 (10), 105 (26), 93 (21), 77 (51), 65 (62), 60 (52), 43 (100). Anal. Calcd. for C_28_H_18_N_4_O_4_S (506.53): C, 66.39; H, 3.58; N, 11.06. Found: C, 66.04; H, 3.21; N, 10.86%.

##### (3-(Furan-3-yl)isoxazol-4-yl)(5-methyl-1-(4-nitrophenyl)-3-(thiophen-2-yl)-1*H*-pyrazol-4-yl)methanone (**13b**)

Orange solid, mp 209–211 °C; IR (KBr) ν_max_ 1598 (C=N), 1664 (C=O), 2925, 3107 (C–H) cm^−1^; ^1^H NMR (DMSO-*d*_*6*_) *δ* 2.34 (s, 3H, CH_3_), 7.13–8.61 (m, 10H, Ar–H), 9.23 (s, 1H, pyrazole-H5); MS m/z (%) 446 (M^+^, 2), 392 (100), 349 (43), 317 (23), 285 (11), 234 (16), 191 (16), 172 (20), 130 (26), 102 (26), 77 (69). Anal. Calcd. for C_22_H_14_N_4_O_5_S (446.44): C, 59.19; H, 3.16; N, 12.55. Found: C, 59.50; H, 2.80; N, 12.17%.

##### Alternate synthesis of **6a** and **13a**

Equimolar amounts of enaminone **9** (0.382 g, l mmol) and hydrazonoyl halide **3a** or hyroximoyl chloride **10a** (1 mmol) in dry toluene (15 mL) containing an equivalent amount of triethylamine (0.5 mL) was refluxed in microwave oven at 500 W and 150 °C for 6 min., gave products identical in all respects (mp, mixed mp and IR spectra) with compounds **6a** and **13a**, respectively.

##### Synthesis of thiazoles **18a**–**c** and ***21a***, ***b****and thiadiazoles****23a***, ***b****: Method A*

To a stirred solution of acetyl pyrazole **1** (0.327 g, 1 mmol), thiosemicarbazide **16** (0.091 g, 1 mmol) and the appropriate hydrazonoyl halides **3a**, **b**, **e** or **3c**, **f** or **3d**, **g** (1 mmol) in dioxane (15 mL), an equivalent amount of triethylamine (0.05 mL) was added. The reaction mixture was heated under reflux for 4–8 h (monitored through TLC). Excess of solvent was removed under reduced pressure and the reaction mixture was triturated with MeOH. The product separated was filtered, washed with MeOH, dried and recrystallized from the proper solvent to give thiazoles **18a**–**c** and **21a**, **b** and thiadiazoles **23a**, **b**, respectively.

##### Method B

Repetition of the same reactions of method A with heating in microwave oven at 500 W and 150 °C for 4–10 min., gave products identical in all respects with those separated from method A. The products **18a**–**c**, **21a**, **b** and **23a**, **b** together with their physical constants are listed below.

##### 4-Methyl-2-(2-(1-(5-methyl-1-(4-nitrophenyl)-3-(thiophen-2-yl)-1*H*-pyrazol-4-yl)ethylidene) hydrazinyl)-5-(phenyldiazenyl)thiazole (**18a**)

Orange solid, mp 219–220 °C; IR (KBr) ν_max_ 1600 (C=N), 2974 (C–H), 3396 (NH) cm^−1^; ^1^H NMR (DMSO-*d*_*6*_) *δ* 2.32 (s, 3H, CH_3_), 2.36 (s, 3H, CH_3_), 2.48 (s, 3H, CH_3_), 6.99–7.93 (m, 12H, Ar–H), 10.65 (s, 1H, NH); ^13^C-NMR (DMSO-*d*_6_): *δ* 9.2, 12.5, 24.6 (CH_3_), 114.5, 121.4, 123.1, 125.2, 126.3, 127.0, 127.9, 128.1, 128.5, 128.9, 135.3, 140.4, 140.9, 143.1, 144.1, 145.3, 145.79, 153.3, 163.4 (Ar–C and C=N); MS m/z (%) 542 (M^+^, 6), 432 (16), 253 (13), 138 (11), 106 (69), 90 (12), 78 (100), 64 (11), 51 (34). Anal. Calcd. for C_26_H_22_N_8_O_2_S_2_ (542.64): C, 57.55; H, 4.09; N, 20.65. Found: C, 57.87; H, 3.70; N, 20.35%.

##### 4-Methyl-2-(2-(1-(5-methyl-1-(4-nitrophenyl)-3-(thiophen-2-yl)-1*H*-pyrazol-4-yl)ethylidene) hydrazinyl)-5-(p-tolyldiazenyl)thiazole (**18b**).

Orange solid, mp 226–228 °C; IR (KBr) ν_max_ 1600 (C=N), 2924 (C–H), 3438 (NH) cm^−1^; ^1^H NMR (DMSO-*d*_*6*_) *δ* 2.17 (s, 3H, CH_3_), 2.32 (s, 3H, CH_3_), 2.36 (s, 3H, CH_3_), 2.47 (s, 3H, CH_3_), 6.99–7.89 (m, 11H, Ar–H), 10.65 (s, 1H, NH); ^13^C-NMR (DMSO-*d*_6_): *δ* 12.0, 14.3, 15.7, 26.8 (CH_3_), 105.3, 111.5, 114.9, 116.3, 117.9, 119.8, 120.8, 122.2, 126.4, 126.6, 127.9, 128.1, 131.9, 132.6, 137.6, 141.7, 142.1, 142.3, 170.2 (Ar–C and C=N); MS m/z (%) 556 (M^+^, 18), 431 (18), 314 (25), 251 (43), 193 (32), 166 (29), 152 (43), 136 (20), 119 (45), 104 (67), 90 (68), 75 (100), 62 (55), 52 (28), 41 (41). Anal. Calcd. for C_27_H_24_N_8_O_2_S_2_ (556.66): C, 58.26; H, 4.35; N, 20.13. Found: C, 58.58; H, 4.05; N, 19.80%.

##### 5-((4-Chlorophenyl)diazenyl)-4-methyl-2-(2-(1-(5-methyl-1-(4-nitrophenyl)-3-(thiophen-2-yl)-1*H*-pyrazol-4-yl)ethylidene)hydrazinyl)thiazole (**18c**)

Orange solid, mp 232–235 °C; IR (KBr) ν_max_ 1598 (C=N), 2922 (C–H), 3436 (NH) cm^−1^; ^1^H NMR (DMSO-*d*_*6*_) *δ* 2.32 (s, 3H, CH_3_), 2.36 (s, 3H, CH_3_), 2.47 (s, 3H, CH_3_), 6.99–7.93 (m, 11H, Ar–H), 10.65 (s, 1H, NH); ^13^C-NMR (DMSO-*d*_6_): *δ* 12.2, 19.1, 24.7 (CH_3_), 120.3, 125.1, 125.3, 125.4, 127.0, 127.1, 127.2, 128.2, 128.4, 134.3, 140.3, 140.4, 143.9, 144.1, 144.2, 145.5, 146.3, 146.4, 170.4 (Ar–C and C=N); MS m/z (%) 579 (M^+^+2, 2), 577 (M^+^, 5), 548 (7), 378 (14), 333 (11), 271 (100), 211 (20), 181 (20), 153 (18), 118 (16), 104 (66), 94 (36), 77 (52), 69 (36), 57 (37). Anal. Calcd. for C_26_H_21_N_8_ClO_2_S_2_ (577.08): C, 54.11; H, 3.67; N, 19.42. Found: C, 54.44; H, 3.35; N, 19.12%.

##### Synthesis of 2-(1-(5-methyl-1-(4-nitrophenyl)-3-(thiophen-2-yl)-1*H*-pyrazol-4-yl)ethylidene) hydrazinecarbothioamide (**19**)

Amixture of acetyl pyrazole **1** (3.27 g, 10 mmol) and thiosemicarbazide **16** (0.91 g, 10 mmol) in ethanol (20 mL) containing catalytic amounts of concentrated HCl was refluxed in microwave oven at 500 W and 150 °C for 6 min., then left to cool to room temperature. The precipitated product was filtered off, washed with ethanol, and dried. Recrystallization from acetic acid afforded thiosemicarbazone **19** as yellow solid, (78% yield), mp 212–215 °C; IR (KBr) ν_max_ 1596 (C=N), 2926 (C–H), 3157, 3241, 3388 (NH and NH_2_) cm^−1^; ^1^H NMR (DMSO-*d*_*6*_) *δ* 2.17 (s, 3H, CH_3_), 2.34 (s, 3H, CH_3_), 7.10 (t, *J* = 1.2 Hz, 1H, thiophene-H), 7.23 (d, *J* = 1.2 Hz, 1H, thiophene-H), 7.56 (d, *J* = 1.2 Hz, 1H, thiophene-H), 7.86 (d, *J* = 8.8 Hz, 2H, Ar–H), 8.20 (s, 2H, NH_2_), 8.38 (d, *J* = 8.8 Hz, 2H, Ar–H), 10.28 (s, 1H, NH); MS m/z (%) 400 (M^+^, 8), 322 (21), 284 (30), 211 (18), 176 (24), 150 (26), 130 (25), 112 (29), 105 (71), 97 (40), 83 (45), 69 (63), 57 (62), 43 (100). Anal. Calcd. for C_17_H_16_N_6_O_2_S_2_ (400.48): C, 50.98; H, 4.03; N, 20.98. Found: C, 51.30; H, 3.73; N, 20.65%.

##### 2-(2-(1-(5-Methyl-1-(4-nitrophenyl)-3-(thiophen-2-yl)-1*H*-pyrazol-4-yl)ethylidene) hydrazinyl)-5-(2-phenylhydrazono)thiazol-4(5*H*)-one (**21a**)

Orange solid, mp 203–205 °C; IR (KBr) ν_max_ 1600 (C=N), 1680 (C=O), 2932 (C–H), 3211, 3420 (2NH) cm^−1^; ^1^H NMR (DMSO-*d*_*6*_) *δ* 2.24 (s, 3H, CH_3_), 2.42 (s, 3H, CH_3_), 7.12–7.92 (m, 12H, Ar–H), 9.82 (s, 1H, NH), 10.27 (s, 1H, NH); ^13^C-NMR (DMSO-*d*_6_): *δ* 12.1, 23.2 (CH_3_), 112.6, 120.9, 125.3, 125.6, 125.9, 127.0, 127.3, 127.8, 128.2, 128.4, 134.3, 140.2, 140.4, 143.1, 144.7, 145.2, 155.5, 160.1 (Ar–C and C=N), 175.4 (C=O); MS m/z (%) 544 (M^+^, 3), 367 (18), 267 (15), 194 (17), 177 (18), 129 (25), 115 (29), 102 (38), 91 (39), 79 (35), 72 (93), 60 (100), 43 (71). Anal. Calcd. for C_25_H_20_N_8_O_3_S_2_ (544.61): C, 55.13; H, 3.70; N, 20.58. Found: C, 55.44; H, 3.40; N, 20.25%.

##### 2-(2-(1-(5-Methyl-1-(4-nitrophenyl)-3-(thiophen-2-yl)-1*H*-pyrazol-4-yl)ethylidene) hydrazinyl)-5-(2-(*p*-tolyl)hydrazono)thiazol-4(5*H*)-one (**21b**)

Orange solid, mp 201–203 °C; IR (KBr) ν_max_ 1596 (C=N), 1675 (C=O), 2920, 2978 (C–H), 3272, 3419 (2NH) cm^−1^; ^1^H NMR (DMSO-*d*_*6*_) *δ* 2.28 (s, 3H, CH_3_), 2.35 (s, 3H, CH_3_), 2.48 (s, 3H, CH_3_), 6.94–8.43 (m, 11H, Ar–H), 10.51 (s, 1H, NH), 10.54 (s, 1H, NH); ^13^C-NMR (DMSO-*d*_6_): *δ* 13.5, 14.5, 21.1 (CH_3_), 112.0, 114.9, 116.3, 117.5, 119.5, 122.2, 125.3, 126.6, 128.0, 129.8, 136.5, 137.4, 138.4, 142.1, 148.2, 151.8, 154.5, 160.1 (Ar–C and C=N), 173.5 (C=O); MS m/z (%) 558 (M^+^, 2), 536 (11), 457 (61), 423 (12), 396 (27), 284 (44), 212 (45), 187 (51), 158 (22), 145 (36), 115 (57), 95 (41), 65 (100), 51 (28). Anal. Calcd. for C_26_H_22_N_8_O_3_S_2_ (558.63): C, 55.90; H, 3.97; N, 20.06. Found: C, 56.20; H, 3.65; N, 19.70%.

##### 2-((1-(5-Methyl-1-(4-nitrophenyl)-3-(thiophen-2-yl)-1*H*-pyrazol-4-yl)ethylidene)hydrazono)-3,5-diphenyl-2,3-dihydro-1,3,4-thiadiazole (**23a**)

Orange solid, mp 195–197 °C; IR (KBr) ν_max_ 1591 (C=N), 2924, 3105 (C–H) cm^−1^; ^1^H NMR (DMSO-*d*_*6*_) *δ* 2.18 (s, 3H, CH_3_), 2.43 (s, 3H, CH_3_), 7.09–8.42 (m, 17H, Ar–H); ^13^C-NMR (DMSO-*d*_6_): *δ* 12.1, 24.7 (CH_3_), 113.6, 120.3, 122.1, 125.3, 125.9, 126.0, 127.5, 127.8, 128.2, 128.4, 130.2, 133.5, 134.3, 135.3, 137.3, 140.4, 143.1, 144.4, 145.5, 146.3, 146.4, 159.4 (Ar–C and C=N); MS m/z (%) 577 (M^+^, 6), 492 (36), 441 (20), 356 (30), 327 (59), 269 (42), 177 (57), 121 (51), 103 (100), 77 (77), 55 (72), 42 (30). Anal. Calcd. for C_30_H_23_N_7_O_2_S_2_ (577.68): C, 62.37; H, 4.01; N, 16.97. Found: C, 62.68; H, 3.70; N, 16.62%.

##### 2-((1-(5-Methyl-1-(4-nitrophenyl)-3-(thiophen-2-yl)-1*H*-pyrazol-4-yl)ethylidene) hydrazono)-3-(4-nitrophenyl)-5-(thiophen-3-yl)-2,3-dihydro-1,3,4-thiadiazole (**23b**)

Orange solid, mp 209–210 °C; IR (KBr) ν_max_ 1693 (C=N), 2954 (C–H) cm^−1^; ^1^H NMR (DMSO-*d*_*6*_) *δ* 2.18 (s, 3H, CH_3_), 2.27 (s, 3H, CH_3_), 7.10–8.42 (m, 14H, Ar–H); MS m/z (%) 628 (M^+^, 7), 561 (11), 510 (31), 441 (20), 360 (26), 313 (24), 284 (78), 270 (52), 190 (26), 152 (100), 105 (63), 89 (30), 63 (39). Anal. Calcd. for C_28_H_20_N_8_O_4_S_3_ (628.70): C, 53.49; H, 3.21; N, 17.82. Found: C, 53.81; H, 2.90; N, 17.51%.

##### Alternate synthesis of thiazole **18a** and **21a**

Equimolar amounts of thiosemicarbazone **19** (0.400 g, l mmol) and hydrazonoyl chloride **3a** or **3c** (1 mmol) in dioxane (15 mL) containing an equivalent amount of triethylamine (0.05 mL) was refluxed in microwave oven at 500 W and 150 °C for 3 min., gave product identical in all respects (mp, mixed mp and IR spectra) with compounds **18a** and **21a**, respectively.

### Biological activity

#### Anticancer activity

The cytotoxic evaluation of the synthesized compounds was carried out at the Regional Center for Mycology and Biotechnology at Al-Azhar University, Cairo, Egypt according to the reported method [49].

## Conclusion

In our present work, we herein present an efficient regioselective synthesis of novel 4-heteroaryl-pyrazoles, which have not been reported *hitherto* in a multicomponent synthesis under microwave irradiation. The structures of the newly synthesized compounds were established on the basis of spectroscopic evidences and their synthesis by alternative methods. The in vitro growth inhibitory activity of the synthesized compounds against hepatocellular carcinoma (HepG-2) and human lung cancer (A-549) cell lines were investigated in comparison with Cisplatin reference drug as a standard drug using MTT assay and the results revealed promising activities of six compounds.

## Abbreviations

A-549: human lung cancer
HepG2: human hepatocellular carcinoma
EtOH: ethanol
mp: melting point
TEA: triethylamine
IR: infra-red
ATCC: American type culture collection
TLC: thin layer chromatography
